# Quantifying Physical Activity in Young Children Using a Three-Dimensional Camera

**DOI:** 10.3390/s20041141

**Published:** 2020-02-19

**Authors:** Aston K. McCullough, Melanie Rodriguez, Carol Ewing Garber

**Affiliations:** 1 Department of Biobehavioral Sciences, Teachers College, Columbia University, New York, NY 10027, USA; mpr2146@tc.columbia.edu (M.R.); ceg2140@tc.columbia.edu (C.E.G.); 2 Department of Music & Dance, College of Humanities & Fine Arts, University of Massachusetts Amherst, Amherst, MA 01003, USA

**Keywords:** machine learning, algorithms, computer vision systems, play

## Abstract

The purpose of this study was to determine the feasibility and validity of using three-dimensional (3D) video data and computer vision to estimate physical activity intensities in young children. Families with children (2–5-years-old) were invited to participate in semi-structured 20-minute play sessions that included a range of indoor play activities. During the play session, children’s physical activity (PA) was recorded using a 3D camera. PA video data were analyzed via direct observation, and 3D PA video data were processed and converted into triaxial PA accelerations using computer vision. PA video data from children (*n* = 10) were analyzed using direct observation as the ground truth, and the Receiver Operating Characteristic Area Under the Curve (AUC) was calculated in order to determine the classification accuracy of a Classification and Regression Tree (CART) algorithm for estimating PA intensity from video data. A CART algorithm accurately estimated the proportion of time that children spent sedentary (AUC = 0.89) in light PA (AUC = 0.87) and moderate-vigorous PA (AUC = 0.92) during the play session, and there were no significant differences (*p* > 0.05) between the directly observed and CART-determined proportions of time spent in each activity intensity. A computer vision algorithm and 3D camera can be used to estimate the proportion of time that children spend in all activity intensities indoors.

## 1. Introduction

The health-enhancing benefits of physical activity (PA) in early childhood (ages 2–5-years-old) have been widely reported [1,2] and evidence suggests that social and contextual factors, such as proximity to others, may influence PA behaviors in young children [3,4,5]. To date, multiple wearable sensors (e.g., accelerometers and GPS devices) have been used simultaneously to provide social and contextual information alongside objective estimates of children’s daily PA volumes [4]. However, the cost of such a multi-sensor measurement approach may be a limitation, and the use of multiple wearable sensors during free-living activities may increase participant burden especially in young children. 

As an alternative method for dynamically measuring PA and social-contextual signals, studies have shown that video data can be processed using computer vision algorithms to extract information about physical activity behaviors within a given context [6]. Computer vision uses an array of techniques from fields such as engineering and machine learning to extract meaningful information (e.g., facial features and hand gestures) from digital images including video [7]. While a small number of studies have used custom computer vision algorithms to convert video-recorded PA behaviors into quantifiable PA signals [6,8,9,10,11], no study has validated such a method for estimating PA volumes and intensities in young children from these signals. A recent report pointed toward the potential benefits of using computer vision, rather than wearable sensors, to measure children’s physical activity while indoors at school [12]. The proof of concept study showed that total vector magnitude signals extracted from video frames were moderately positively correlated with wearable sensor-derived triaxial physical activity accelerations in children and an adult. Study findings revealed that vector magnitude data extracted from video could possibly be used to measure children’s PA within indoor settings. However, the report states that more discriminating algorithms for analyzing vector magnitude data in videos may be required than those employed in their feasibility analyses.

Of the available studies that have used computer vision to specifically estimate PA volumes [6,8,10,11,12], few have expressly calibrated an algorithm to quantify and classify activity data in children [8]. A study of 10-year-old children appears to be the first to demonstrate the feasibility of using a ceiling-mounted camera to automatically derive estimates of PA velocities in bidimensional space [8]. Concurrently, a feasibility study demonstrated the potential application of three-dimensional (3D) cameras to measure PA [9]; however, PA was only analyzed in one of the available three dimensions and the uniaxial activity signals were not calibrated against a criterion measure of physical activity. A recent report in adults revealed that computer vision algorithms can also accurately estimate time spent in PA outdoors when compared to accelerometry [6], but direct application of these findings may be limited to use in adults alone given that energy expenditure profiles, as they relate to physical activity, change over time as a function of biological maturation [13]. As such, further video-based physical activity calibration studies are needed in young children to determine the practical use of 3D video for measuring PA at this developmental stage.

While the feasibility and validity of using computer vision to estimate activity volumes and intensities appears promising [6,8,9,10,11] and the use of such technology for estimating children’s PA while indoors has been presented as a logical step forward [12], little is known about the use of computer vision methods to measure PA in young children. Given the lack of validated methods for assessing young children’s PA indoors from video data via automated processes, and aiming toward developing more robust contextual models of physical activity behavior, the need for further research on 3D video data calibration for estimating physical activity levels and volumes in young children is clear. Therefore, the purpose of this study was to determine the feasibility and validity of using 3D- camera-derived physical activity intensity vector magnitude signals to estimate physical activity intensities indoors in a sample of 2–5-year-old children.

## 2. Materials and Methods

### 2.1. Sample

Families with 2–5-year-old children were recruited from community centers, preschools, early childhood centers, daycares, and a hospital located within a major urban center via flyers, emails, and word-of-mouth. Children for whom engaging in moderate-vigorous physical activity would present any concerns for safety due to existing medical conditions were excluded from participating in the study. The presence of exclusion criteria was confirmed via parental report at the time of recruitment and were confirmed during an orientation session. Informed consent was obtained by one or both parents for *N* = 11 children, and the study was approved by the Institutional Review Board at both Columbia University Medical Center and Teachers College, Columbia University.

### 2.2. Play Session Protocol

After providing informed consent, families were scheduled to attend a 20-minute indoor play session. The play session took place on a 100 square foot padded play area located within the laboratory. Prior to beginning the play session, parents were informed that the target physical activity behaviors for children to perform were quiet play, walking, climbing, running, and jumping. Children and their parents were then invited to play freely with various affordances for movement (e.g., toy cars, blocks, risers, bubbles) for the first 10 minutes of the play session. If all of the target behaviors were performed within the first 10 minutes of the play session, families were invited to continue engaging in semi-structured play for the remaining 10 minutes. Otherwise, if a given behavior was not performed within the first 10 minutes of the play session, a member of the research team introduced various games (e.g., “tag”) and pretend play scenarios into the play session in order to encourage children to perform the target behavior.

### 2.3. Measures

In order to assess physical activity during an indoor play session, sociodemographic, anthropometric, and behavioral data were collected:

#### 2.3.1. Sociodemographic Characteristics

Parents were asked to complete a questionnaire that included items on children’s sex and age.

#### 2.3.2. Anthropometric Measurements

Children’s height was measured in meters (m) to the nearest 0.1m using a stadiometer, and weight was measured in kilograms (kg) to the nearest 0.1kg using a calibrated scale. Body Mass Index (BMI) was calculated as kg/m^2^, and children’s BMI percentiles were determined according to the reference values provided by the Centers for Disease Control [14].

#### 2.3.3. Physical Activity

In order to validate a triaxial physical activity intensity classifier for 3D video data, physical activity was concurrently measured during the play session using Microsoft’s Kinect for Windows and also by direct observation (i.e., the ground truth).

##### Microsoft’s Kinect

Children’s play sessions were video recorded using Microsoft’s Kinect (MSK) for Windows (v1). MSK is a low-cost, portable 3D camera equipped with a color and infrared-depth sensor [7], and the reliability of the device has been established in prior research [15]. Data from both the color and infrared sensors were acquired and processed using a custom image acquisition and processing algorithm developed by the author (A.K.M.) in MATLAB R2017b (The MathWorks, Inc., Natick, MA), as described below:

In studies of wearable physical activity monitors, triaxial acceleration data simultaneously reflect displacement (i.e., movements or perturbations) for a given body segment along the mediolateral, vertical, and anteroposterior planes over time [16]. A prior study revealed that the overall motion vector magnitude detected within a series of video frames could potentially be used to estimate PA within a group while indoors [12]. For making individual-level PA estimates, it follows that pixel values acquired from the 3D video data, as described below, were used to extract triaxial vector magnitude PA signals along the mediolateral (*x*) and vertical (*y*) planes, and the depth sensor intensity values were used for the anteroposterior (*z*) plane.

**Figure 1:**
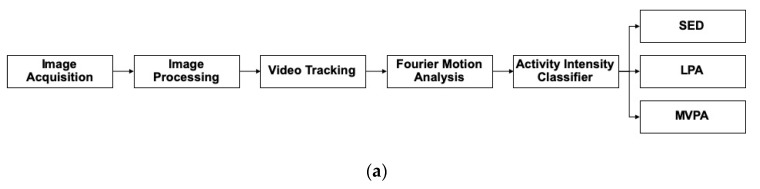

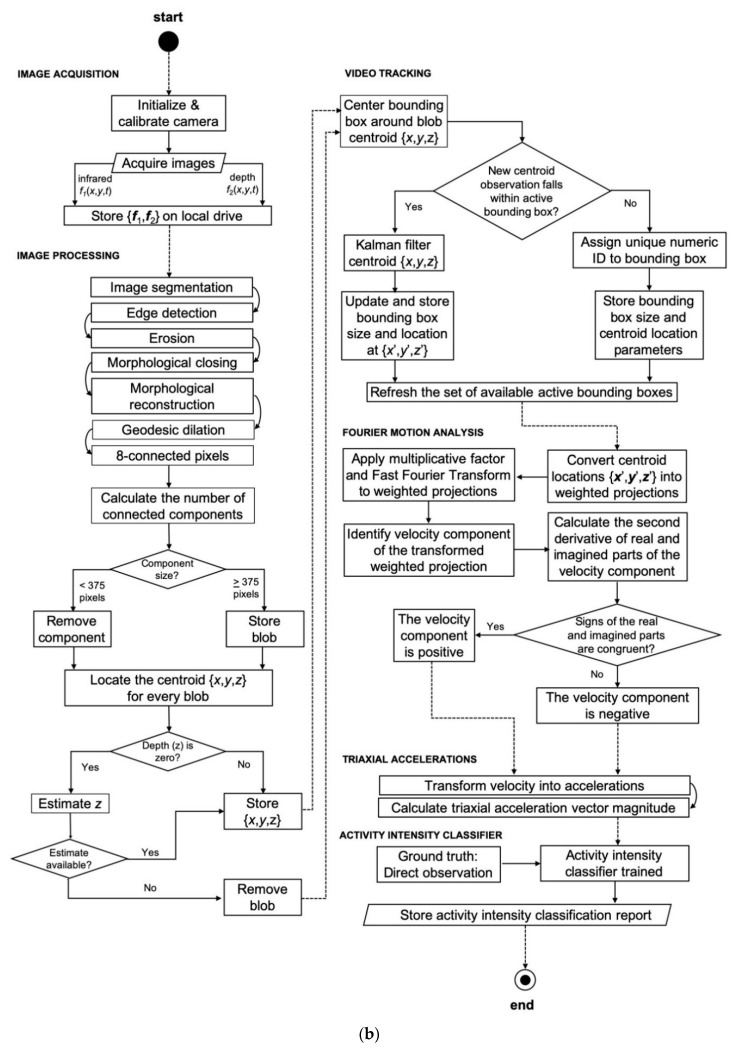
Physical activity video data analysis overview for detecting young children’s physical activity intensities from three-dimensional video data. Note: (**a**,**b**) display the image acquisition, signal processing, and analysis workflow used to classify 2–5-year-old children’s physical activity intensities from video data. Abbreviations: sedentary behavior (SED), light physical activity (LPA), moderate-vigorous physical activity (MVPA).

In order to classify children’s PA intensities from videos (see Figure 1), multi-phased analyses of PA video data were conducted across several stages that included the following: 1) image acquisition, 2) image processing, 3) video tracking, and 4) Fourier motion analysis. At each stage of the analysis, bespoke algorithms were implemented, as described further below, in order to ultimately convert the 3D video signals into triaxial physical activity accelerations.

##### Image Acquisition

All play session image data were quantized as 16-bit unsigned integer color images *f*(*x*, *y*, *t*) and were sampled using the infrared camera. Each video frame was sampled at a resolution of 640 × 480 pixels at 30 Hz, where *x* and *y* represent respective row and column pixel coordinates and *t* represents the time of image acquisition for each frame. Sampling and quantization parameters were identical for the depth sensor; however, pixel values at a given coordinate (*x*, *y*) represent the sensor-determined distance between the sensor and the object in millimeters. The image acquisition routine collected data from the infrared and depth sensors simultaneously, and time was measured in milliseconds using the local CPU time.

**Figure 2:**
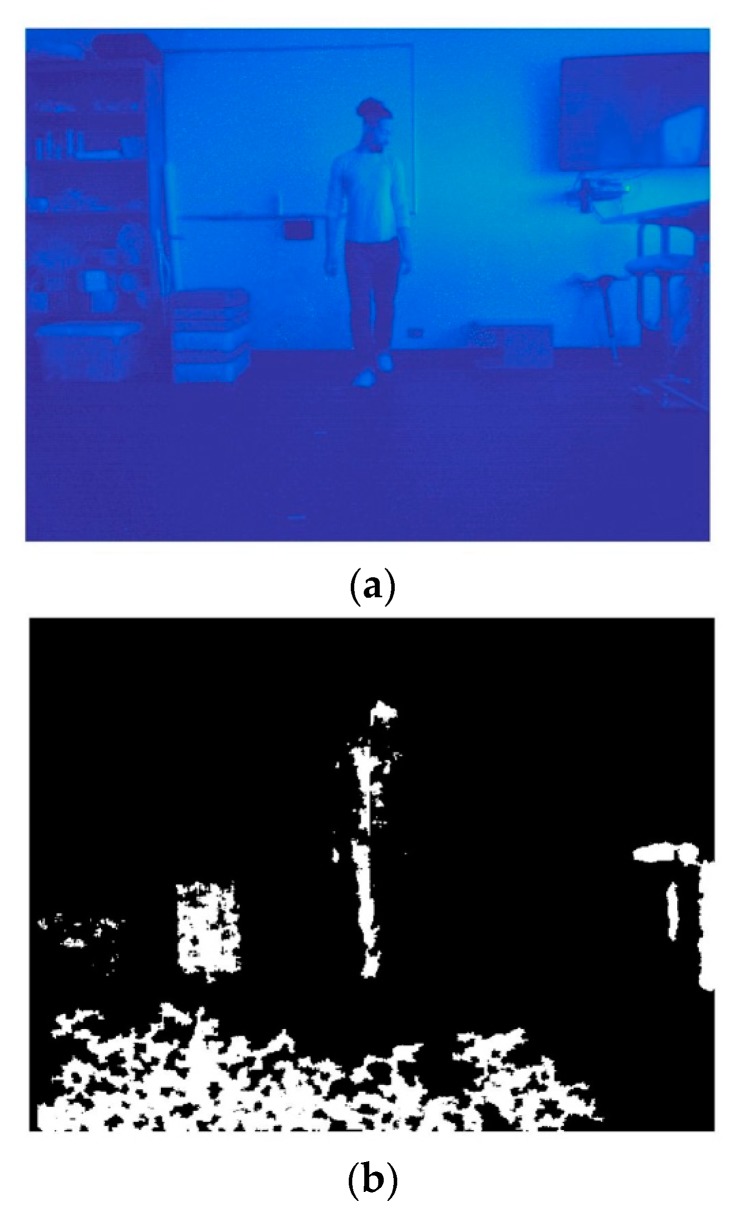

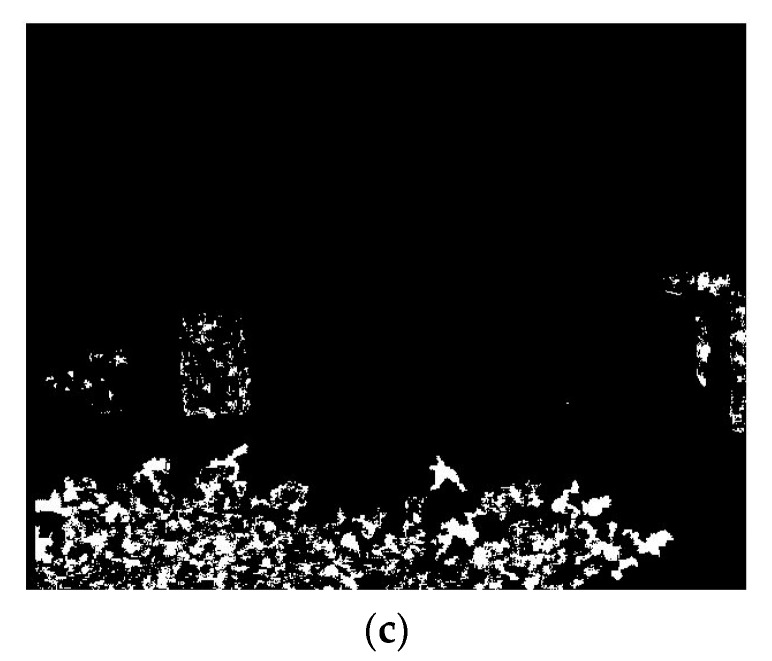
Frames of physical activity acquired with the Kinect infrared (**a**) and depth sensors (**b**,**c**). Note: Figure 2 shows results of the image acquisition algorithm (**a**,**b**) and also that the object of interest was unable to be quantized by the depth sensor when the object was flush against the wall (**c**).

As shown in Figure 2, the image acquisition algorithm captured physical activity data from both the infrared (Figure 2a) and depth (Figure 2b) sensors. Data frames were visually inspected to determine the performance of each of the sensors when capturing physical activity data at various locations in the environment. While the infrared camera consistently collected data across all frames, the depth sensor was unable to quantize object depth when the subject was flush against the wall (Figure 2c). Given that values from the anteroposterior dimension are required in order to compute triaxial acceleration values, frames initially lacking depth data for the object of interest required additional consideration, as described further within the algorithm. *Calibration*—Prior to collecting data during the play sessions, an object of known height (1.75 m) was placed at various distances from the sensor. The pixel coordinates corresponding to the upper- and lower-most edges of the object were manually collected within each frame of interest. The Euclidean distance for each respective pair of points was calculated, and the depth sensor pixel intensity was conserved at the centroid location for the object, thus yielding the object height in pixels and its given distance from the sensor. The constant height of the object was then divided by the Euclidean distance-determined object height, thus yielding an array of scaling values in units of m/pixel. A line of best fit between scaling values (m/pixel) and pixel intensity values (depth) was evaluated:

(1) $$\frac{m}{pixel}=1.5*10^{-06}*\left(depth\right)+6.4*10^{-04}$$

and the resultant formula (Equation (1)) was used within a Fourier motion analyses, as described below, to determine the required m/pixel scaling value for each depth pixel intensity value that was returned for any given centroid location.

##### Image Processing

**Figure 3:**
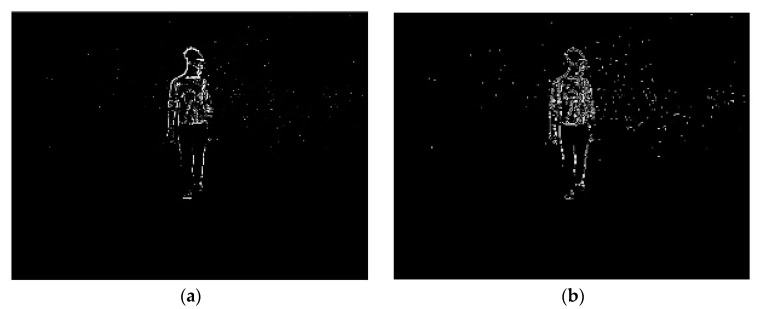

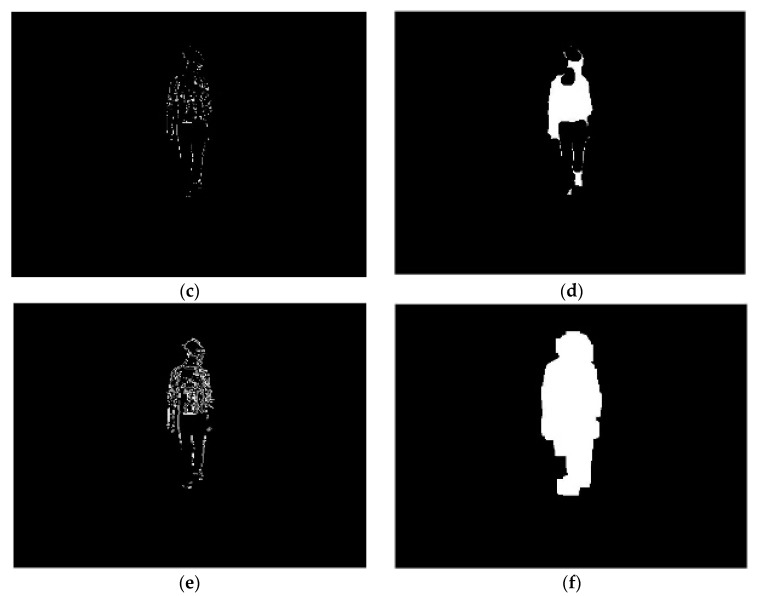
Iterative image processing of a physical activity frame acquired from the Kinect infrared sensor. Note: Figure 3 shows the iterative results of taking the difference image (**a**), Sobel edge detection (**b**), regional pixel majority (**c**), morphological closing (**d**), reconstruction (**e**), and geodesic dilation followed by the filling of any holes in the object (**f**).

For the purposes of image segmentation, difference images (shown in Figure 3a) between consecutive infrared video frames were calculated (Equation (2)),

(2) $$d_{ij}\left(x,y\right)=\;\{\begin{array}{cc} 1 & \mathrm{if}\;\left|f\left(x,y,t_{i}\right)-\;f\left(x,y,t_{j}\right)\right|>14000\\ 0 & \mathrm{otherwise} \end{array},$$

where *d*_ij_(*x*, *y*) is the resultant image, *t_i_* denotes the image frame to be differenced, and *t_j_ = t*_*i*−1_ as the reference image frame [17]. Any *d_ij_*(*x*,*y*) with a total number of foreground pixels greater than threshold *T* = 14,000 were iteratively deemed to be unevaluable given that they were too noisy to sufficiently extract objects of interest. In order to enhance object boundary information contained in *d_ij_*(*x*,*y*), a Sobel edge detector was applied in both the horizontal and vertical directions (Figure 3b). Subsequently, the edge-detected image was convolved with a kernel of size 3 × 3 pixels, where the origin in the output image *g*(*x*, *y*) was equal to 1 when the sum of the kernel in *f*(*x*, *y*) > 5 (Figure 3c). As shown in Figure 3d, morphological closing was then applied to the resultant image using a disk (*radius* = 9 pixels) for the purpose of maximizing object shape information contained within the determined boundary.

Figure 3e shows the results of morphological reconstruction using the closed image as the marker and *d_ij_*(*x*,*y*) as the mask. In order to derive a single connected component that delineated the object of interest, geodesic dilation was applied to the reconstructed image using the dilated reconstructed image as a marker, where the structuring element was a square (*width* = 25 pixels). Afterward, holes were filled in the dilated image using 8-connected pixels (Figure 3f). Finally, the object was thinned for the purpose of reducing the size of any residual noise components in the image.

**Figure 4:**
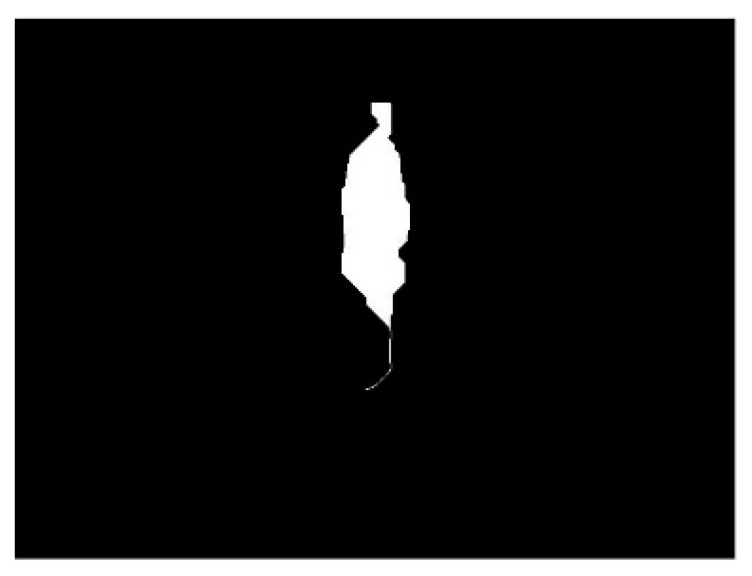
Final infrared physical activity image after image processing, thinning, and masking. Note: Figure 4 shows the resultant blob derived from the combination of image processing and image description (i.e., connected component) algorithms.

The number of connected components in the thinned image were calculated and returned in an output image *z*(*x*, *y*). Any components comprised of <375 pixels were set to zero, and the resultant mask *z*_1_(*x*, *y*) was multiplied by the thinned image. Thus, any additional artifact in the image was removed, as shown in the final product of the algorithm (Figure 4), and any connected component > 375 was conserved as an object of interest. 

**Figure 5:**
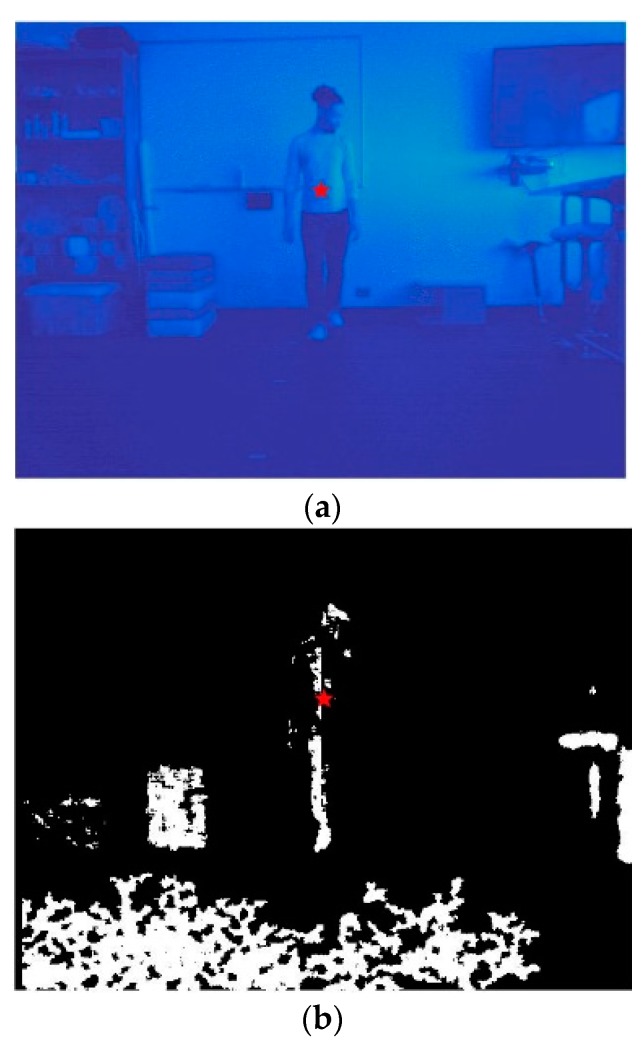
Object centroid superimposed onto infrared (**a**) and depth (**b**) frames. Note: Figure 5 shows infrared image with color remapping (**a**), depth sensor with color axis scaling (**b**), and the object centroid as a red star.

In order to distill a single pixel that represents the position of the object, as needed for further motion analyses, the object centroid was evaluated using the thinned image. Figure 5a shows the enlarged object centroid superimposed on the original infrared image (with updated color mapping) and the depth image (scaled for visibility). As illustrated in the depth sensor image (Figure 5b) and as noted previously (Figure 2c), there is a chance that the depth sensor pixel intensity at the centroid coordinates may be equal to zero due to sensor sensitivity.

**Figure 6:**
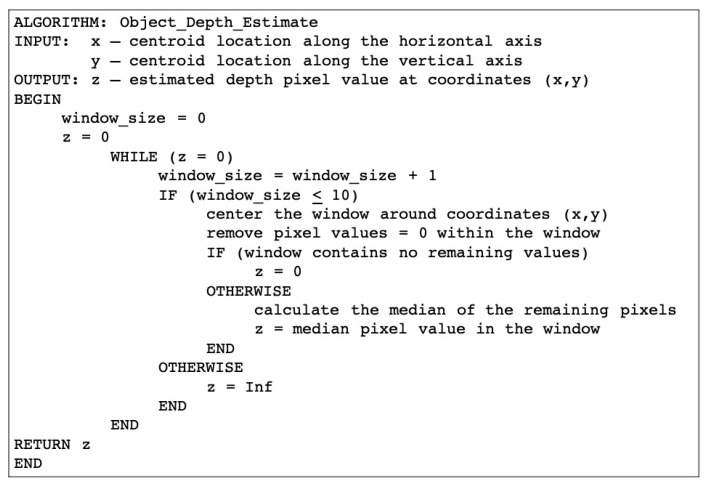
Iterative depth sensor intensity value search algorithm, with increasing window size. Note: Figure 6 outlines an algorithm that searches for the nearest pixel intensity to a given centroid.

To determine if an evaluable depth sensor intensity value was proximal to the centroid coordinates, an increasing window around the centroid was evaluated with a maximum window size of 21 × 21 pixels (Figure 6). Any frame without a depth sensor intensity value was deemed unevaluable within any triaxial acceleration calculations.

##### Video Tracking

**Figure 7:**
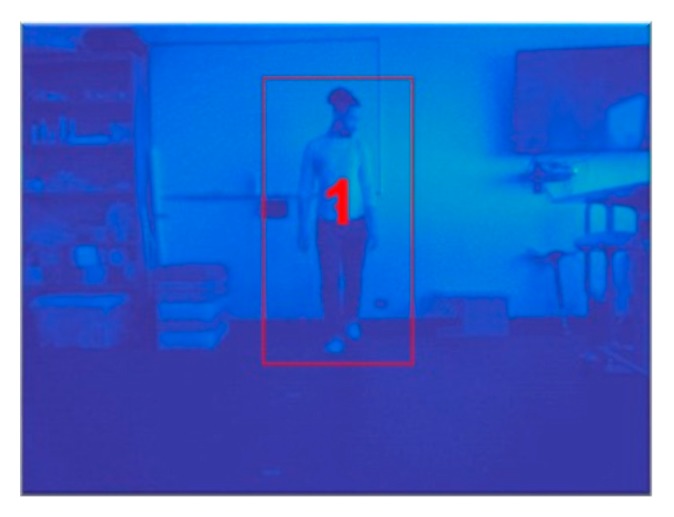
Smoothed centroid location with unique identifier and bounding box. Note: Figure 7 shows the Kalman filtered centroid location, where the centroid location is indicated by the unique numerical identifier (i.e., “1”). A bounding box also appears around the object of interest.

In order to track each object, the height and width of each extracted object of interest (i.e., a blob—see Figure 3) within a given frame were determined, and a bounding box was centered around each blob at its centroid, framing the object of interest. A unique numeric identifier (Figure 7) was assigned to each bounding box, and the time at which the blob was first detected with its assigned numeric identifier was also stored.

**Figure 8:**
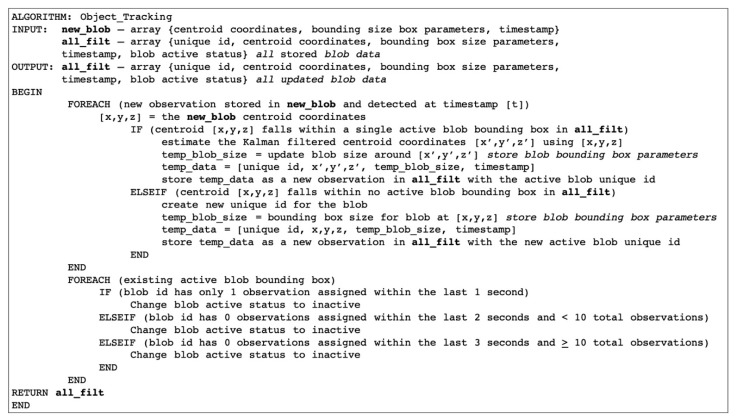
Object tracking algorithm for physical activity video data. Note: Figure 8 shows that the centroid for each detected blob (i.e., object of interest detected within a video recording of physical activity) is assigned a unique identifier and passed to a Kalman filter.

As outlined in Figure 8, the pixel intensity value, centroid location (i.e., coordinates), and bounding box size and location parameters were respectively stored for every detected blob for both the infrared and depth images for each frame. The centroid location was passed to a Kalman filter for signal smoothing [8,18].

Videos were reviewed to confirm that unique identifiers were consistently assigned to the correct child [8]. A member from the research team (AKM) reviewed all videos with unique identifiers and recorded the unique identifier(s) assigned to each child within a given play session. Unique numeric identifiers assigned to parents and research team members who were captured on video during the play sessions were not used for this study.

##### Fourier Motion Analysis

After manually collating all unique identifiers assigned to each child across play sessions, intensity value arrays associated with the respective centroid locations for children in each play session video were used to conduct Fourier motion analyses. For each respective plane of movement (*x*, *y*, *z*), the singular intensity value at a given centroid location was extracted and used to develop a weighted projection [17]. For example, the intensity value given at centroid location (*x*, *y*) in the *M* × *N* image was projected onto a 1-D array of size 1 × *N*, with the pixel value at location *x* for all *x*-axis projections. The same was done for all *y-* and *z*-axis projections. The resultant weighted projections were each multiplied by

(3) $$exp\left(c2\pi a_{1}q\Delta t\right),$$

where *a*_1_ is a positive integer equal to 30 divided by the maximum velocity expected in a given plane, *q* is each value in the projected array, Δ*t* is the relative time interval between frames, and *c* = $\sqrt{-1}$. The maximum velocity expected was 4 m/s in the *x* and *z* planes and was 2.8 m/s in the *y* plane. Following this, the sum of all transformed elements in a given array was calculated. The fast Fourier transform was then computed for each projected array, each of size *K* = 30, where *K* is the relative frame observed during a given second. A peak search over the 30 transformed data points collected at each second revealed the frequency-velocity relationship as the location of the first peak (*Va*_1_) within the signal. Velocity (*V*), defined as the given number of pixels of motion in 30 frames, was then determined by dividing the corresponding frequency value located at the peak location (*Va*_1_) by *a*_1_. To derive the sign of the velocity component, the second derivative of the transformed projection was calculated for the real and imagined components of the values. Where the resultant signs for the real and imaged components were congruent, the velocity was positive, and it was negative otherwise. To convert the velocity into m/s, *V* was multiplied by the results of (Equation (1)), using the observed intensity value from the depth sensor for the object in the given frame. This process was iterated across all planes for all observations.

##### Triaxial Accelerations

Velocity values obtained from Fourier motion analysis were transformed into acceleration (in *g*) using standard methods [19], the Euclidean norm of the acceleration signals was used to calculate the vector magnitude (VM), $\mathrm{VM}=\sqrt{\left(x^{2}+y^{2}+z^{2}\right)}$, and resultant values were multiplied by 1000. All VM data were then aggregated over 5s epochs for each respective play session file. A video of a study member in the laboratory and rendering of triaxial physical activity accelerations with the gravity component subtracted is available in the Supplementary Materials (Video S1).

Review of the data showed that the infrared-depth sensor signal was severely corrupted by noise in one child’s play session, thus this case was removed from the data set. For the remaining children (*n* = 10), triaxial acceleration data that corresponded to the first 5 min of the play session were extracted and used for comparison to direct observation.

##### Children’s Activity Rating Scale (CARS)

As previously noted, direct observation of play session data was used as the ground truth. Unprocessed 2D play session video data that were collected using the infrared camera were converted from *.bin files into 16-bit, 640 × 480 pixel images. Following, each frame was processed in the spatial and frequency domains to remove periodic noise from the color video signals, as well as to improve intensity value contrasts where videos appeared to have too little ambient light exposure. Restored videos were then exported for direct observation analyses as *.avi video files using a custom algorithm developed in MATLAB. Using the restored video recordings, children’s physical activity behaviors were coded using a second-by-second CARS protocol [20,21]. CARS activity intensity categories (i.e., 1 = lying down or sitting; 2 = standing; 3 = walking; 4 = walking, moderate; 5 = running, strenuous activity) were modified to reflect the following four categories: (1) lying down or sitting, (2) standing, (3) walking, (4) running or jumping [20,22]. A final category was also added, (5) not in frame, and any frame with this label was not further analyzed. Two trained coders (AKM and MR) applied the CARS protocol to play session *.avi color videos using a custom computer-based direct observation system and GUI developed in MATLAB by the author (AKM). After coding all videos, the second-by-second data were reintegrated into 5s epochs using a standard weighted average formula [22]. For every 5 s, each activity code within the epoch was multiplied by the frequency of its occurrence in the epoch, and the mean was calculated iteratively. Following, the weighted CARS scores were recoded into the standard physical activity intensity classifications using the following thresholds: sedentary behavior (SED) <2; light physical activity (LPA) 2 to 2.99; and moderate-vigorous physical activity (MVPA) ≥3 [22]. In order to assess inter-rater reliability between CARS coders, the intraclass correlation (ICC) was calculated for *n* = 4 randomly selected 20min play session videos that were coded by both raters. The ICC for the weighted mean CARS scores (*r*_ICC_ = 0.95) showed acceptable agreement between raters.

### 2.4. Statistical Analyses

Descriptive statistics are presented as Mean (Standard Deviation), Median (Interquartile Range), and Frequencies (%(n)), and all data were analyzed in MATLAB. Triaxial VM activity data derived from the computer vision algorithm were used to train a Classification and Regression Tree (CART) algorithm to classify physical activity intensity in young children. For a binary outcome variable, the CART algorithm iteratively partitions a given predictor space into a cascade of binary decisions (i.e., a decision tree) that estimate the target class while reducing the overall model classification error [23]. Thus, target classes for the CART classifier were SED versus LPA/MVPA combined, LPA versus SED/MVPA combined, and MVPA versus SED/LPA combined [22]. A multiclass CART (MCART) algorithm was also implemented, wherein all target classes were simultaneously estimated, the triaxial VM activity data were the sole training feature, and all three of the directly observed activity intensities were targets. In order to determine the accuracy of the CART and MCART classifiers, the respective Receiver Operating Characteristic Area Under the Curve (AUC) and bootstrapped 95% confidence intervals were computed. Sensitivity and specificity were also calculated for both classifiers. Statistical equivalence between the observed and estimated percentages of time spent in each activity intensity were evaluated using nonparametric Two One-Sided Tests (TOST) to determine if the median difference between values (Δ) were <10% and <20% [24], with the significance level established a priori at *α* = 0.05.

## 3. Results

**Figure 9:**
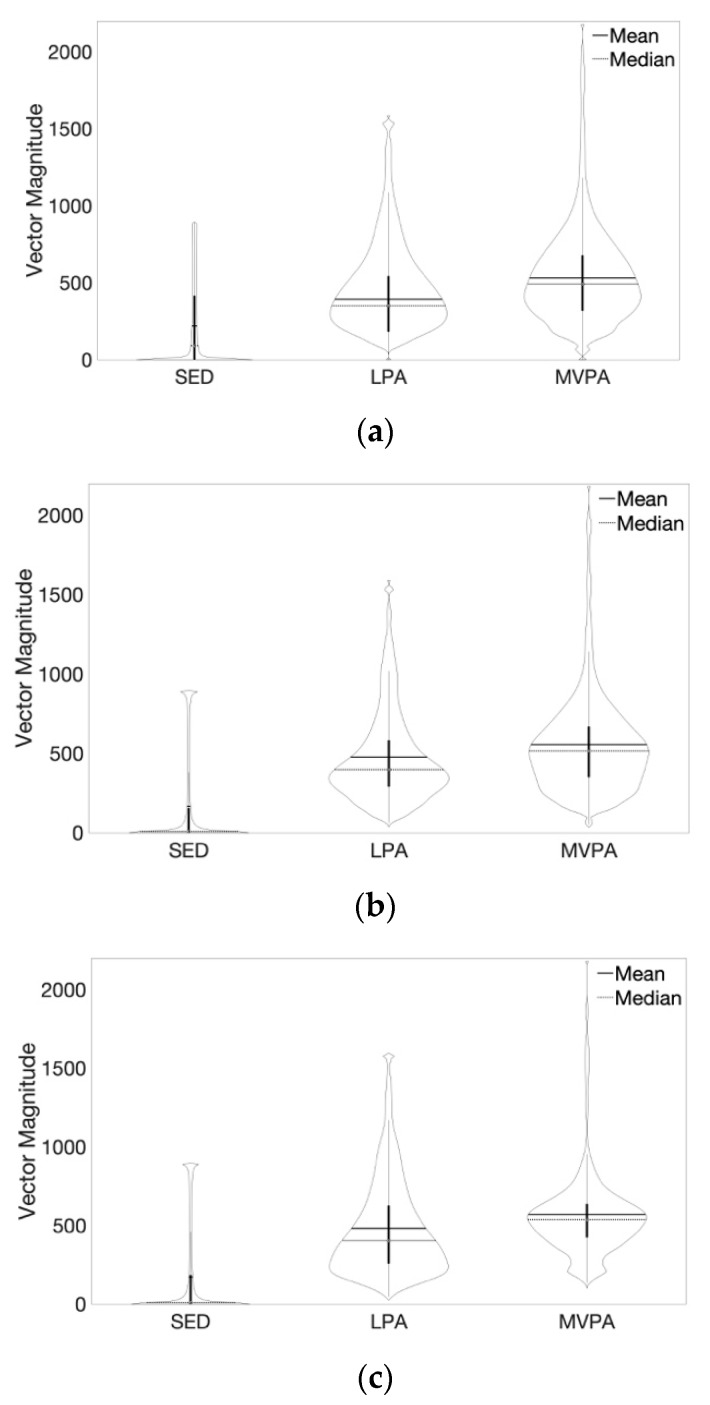
Violin plots of triaxial activity vector magnitude signals collected in young children using a three-dimensional video camera during indoor play. Note: Figure 9 shows the class-specific distributions of physical activity triaxial acceleration vector magnitude signals that were distilled from semi-structured indoor play session video data using a bespoke computer vision algorithm. (**a**) shows the vector magnitude signals for each activity intensity class partitioned given the direct observation-based classification. (**b**,**c**) respectively, show the vector magnitude signals for each activity intensity class partitioned given the CART and Multiclass CART classifiers. Abbreviations: sedentary behavior (SED), light physical activity (LPA), moderate-vigorous physical activity (MVPA), Classification and Regression Tree (CART).

Children were *45*(12) months-old on average, 60% (6) were girls and 40% (4) were boys. BMI percentiles showed that 70% (7) of children were normal weight, 20% (2) were at risk of being overweight, and 10% (1) were overweight. Figure 9 displays class-specific distributions for the video-derived triaxial physical activity vector magnitude signals for each activity intensity, with class partitions determined by direct observation, CART and MCART models, respectively.

**Figure 10:**
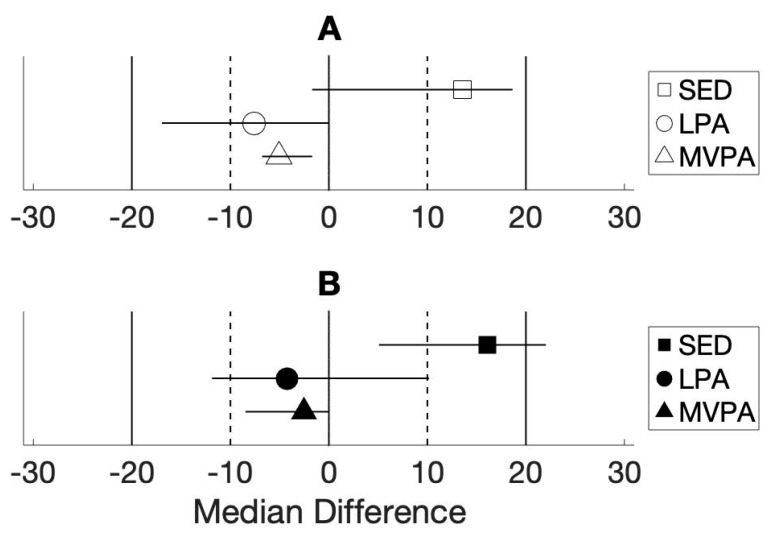
Median differences in the observed and classifier estimated percentages of time spent in each activity intensity. Note: Figure 10 shows the class- and classifier-specific median differences between the observed and classifier-estimated percentages of time spent in sedentary behavior (SED), light physical activity (LPA), moderate-vigorous PA (MVPA), and 90% Confidence Intervals. (**A**,**B**) respectively show differences in the percentage of time spent in each activity intensity, relative to the ground truth, for the CART and Multiclass CART classifiers.

Table 1 and Figure 10 show the performance of the CART-estimated percentages of time spent in each activity intensity as compared to direct observation. Direct observation data showed that children spent the highest percentage of their time during the semi-structured play session in LPA, followed by MVPA and SED. Both the CART and MCART classifiers overestimated the proportion of time that children engaged in SED, and underestimated LPA and MVPA time.

**Figure 11:**
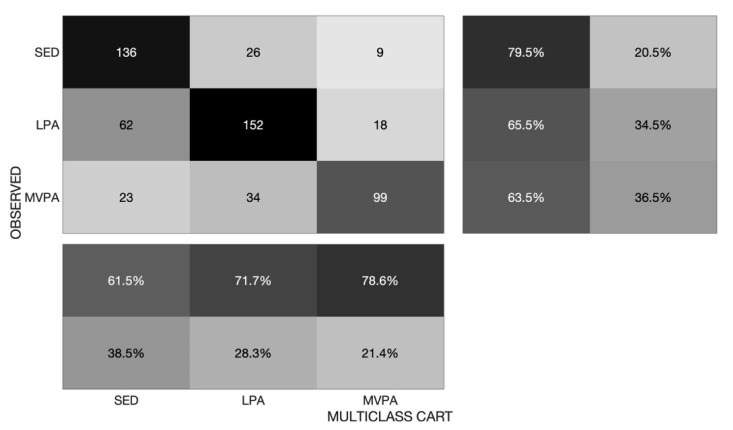
Confusion matrix for the observed versus Multiclass CART classifier-estimated activity intensity classification. Note: Figure 11 shows correctly labeled cases for the Multiclass CART classifier on the diagonal, and mislabeled cases are shown on the off-diagonal. Row- and column-normalized percentages of correctly versus incorrectly labeled classes are shown in the margins.

The CART-estimated activity volumes were statistically equivalent to the directly observed percentages of time, as children engaged in activity within 20% of the ground truth at every intensity level (*p* < 0.05); however, only MVPA volume estimates were statistically equivalent at <10% error. Relative to direct observation, estimates of the percentage of time spent on SED showed ≥20% error for the MCART classifier (*p* > 0.05), LPA estimates were statistically equivalent at <20% error (*p* < 0.05), and MVPA estimates were equivalent at <10% error (*p* < 0.05). The MCART classifier confusion matrix shows that the highest total number of mislabeled classes were directly observed LPA cases that were mislabeled as SED by the classifier, followed by MVPA cases that were mislabeled as LPA (Figure 11).

## 4. Discussion

This study aimed to calibrate a computer vision algorithm to estimate physical activity behavior intensities in young children using a 3D camera. To our knowledge, this is the first study to use computer vision to objectively measure PA intensity in young children. Results showed that triaxial physical activity acceleration signals derived from a 3D camera can be used to accurately estimate children’s physical activity intensity and the relative percentages of time spent in each activity intensity without the use of a wearable sensor. The CART classifier estimated the percentage of time that 2–5-year-olds spent in each activity during an indoor play session within 5–14% of the ground-truth-determined percentages of time. For the MCART classifier, the estimated percentages of time spent in each activity intensity were within 3–16% of the observed percentages of time.

Percentages of time spent in each activity intensity were estimated at the individual-level in our study of 10 children, and participants were allowed to engage in their own choice and sequence of activities during the semi-structured play session with their parents. A prior study in 8 children (10-year-olds) calibrated a video-tracking algorithm to estimate group-level PA intensities using a low-frequency sampling method (i.e., periodic 10s observations) while children played basketball indoors [8]. In a study of 9 adults, activity intensities were estimated at the group level while participants were asked to sit, stand, walk, and jog outdoors using an ecological assessment measurement approach that took periodic activity samples [6]. Comparatively, similar to Koporec and colleagues [11], a continuous sampling approach was used during our semi-structured and indoor play session protocol in young children. Additionally, children’s presentation of the semi-structured activities within sessions was highly dynamic and variable. Thus, our algorithm and physical activity intensity classifiers were specifically calibrated to capture short-burst, multiplanar physical activity behaviors that young children typically exhibit [12,13]. Our study contributes evidence of both the feasibility and validity of using computer vision to analyze individual-level PA behaviors within indoor contexts to this growing area of physical activity measurement research.

A recent proof of concept study proposed the use of the overall motion VM signal from video data as a potential means by which to estimate children’s physical activity volumes while indoors [12]. In lieu of using the motion VM of all pixel movement captured within a series of video frames, our study computed individual-level physical activity VM signals in order to estimate activity volume and intensity in 2–5-year-old children. We found that VM signals between activity classes may overlap for some physical activities, which aligns with wearable sensor studies that have used VM-based measures as the sole signal feature for class estimation [25]. For example, as with robust wearable sensor studies that have used VM-based signals to distinguish SED and non-SED episodes [26], we found that a large degree of children’s motionless standing episodes were mislabeled as SED, though standing is classified as non-SED by definition [27]. This is likely due to the fact that our algorithm derived the activity VM trace from the object centroid, which is similar to collecting acceleration data from a waist-worn wearable sensor. Similarly, paralleling prior wearable sensor studies that have investigated physical activity intensity classification in resistance training paradigms [28], we found that when young children were pushing, or walking while carrying heavier objects in the play space, that these episode were mislabeled in our study as LPA rather than being correctly classified as MVPA [20]. Nevertheless, VM signals in our study provided sufficient information for identifying children’s activity intensities while indoors with good accuracy. Future studies using computer vision to classify activity intensities in children may consider the use of human activity recognition algorithms [29,30,31] to specifically target sedentary behaviors, such as quiet sitting, from motionless non-sedentary activities, such as quiet standing or performing an isometric bodyweight resistance exercise (e.g., a sustained squat). Furthermore, the estimation of object mass from images is a nontrivial problem [32], which requires additional attention in the computer vision physical activity literature with respect to differential activity intensity classification during human-object interactions.

A strength of our study is that 3000 s of video data were used to calibrate the physical activity intensity classifiers across ten 2–5-year-old children using a wide variety of affordances for movement. A prior validation study using computer vision to specifically measure PA intensities in children used a total sample of 1000 s for algorithm calibration as children played basketball indoors [8]. The activity intensity classifiers in our study were trained while children played with a wide variety of affordances for movement ranging in size from a 55-inch-long foam pool noodle to 5-inch-long wooden toy cars. The affordances also ranged in weight from 0.16-ounce silk scarves to a stack of four aerobic steppers that were each ~6 pounds. Thus, our findings offer additional evidence for the use of computer vision–based methods to classify activity intensities in children while indoors and while engaged in various kinds of human-object interaction, as well as human-to-human and independent tasks, with sufficient accuracy.

At the same time, a limitation of this study was that the sample size precluded the inclusion of additional covariates that may help to improve physical activity intensity estimates, such as age. Though studies using accelerometers have shown that activity intensity cut points may be similar for toddlers (2–3-year-olds) and preschool-aged (3–4-year-olds) children at higher activity intensities [22], research also shows that age-specific physical activity classifiers may improve activity estimates for accelerometer-derived data [33]. As such, further research in larger samples is needed to determine if 3D camera-derived physical activity intensity classifiers would benefit from the inclusion of age as a model covariate in young children. The activity classifiers in this study were trained using only indoor physical activity data; therefore, these findings may be limited to similar indoor physical activity measurement environmental contexts. Future studies should continue to develop methods for measuring physical activity in young children across a broader range of contexts using computer vision. Finally, the calibration cut point values and equations used within our bespoke computer vision algorithm were optimized specifically for the 10 foot × 10 foot play area in our laboratory. As such, calibration parameters may require retuning within other indoor spaces given that environmental conditions were constant within our closed indoor space, and controlled analyses on the impact of environmental variability on algorithm performance was outside of our study delimitations.

We also note, though beyond the scope of this paper, that comparing and harmonizing the methodologies implemented across the growing number of physical activity measurement studies using computer vision is necessary for generating databases of generalizable findings. Moreover, additional research is needed to understand if, and to what degree, the available recommendations for early childhood wearable sensor-based physical activity measurement—such as daily wear-time periods (or, for video data, daily time in the camera field of view), analytic epoch length, and activity intensity cut point specificity [34]—are necessary and sufficient within video-based physical activity measurement.

## 5. Conclusions

Young children’s 3D video-derived triaxial physical activity vector magnitude signals can be used to quantify children’s time spent in sedentary behavior, and light or moderate-vigorous physical activity during an indoor play session. However, vector magnitude signals alone may be insufficient for accurately distinguishing the activity intensity of some indoor behaviors. In view of the lower error for the one-against-all activity intensity classifier versus the multiclass classifier, we consider that 3D video-derived vector magnitude data may be sufficient for identifying young children’s time spent in a single target activity intensity. Future studies may use 3D video-derived triaxial vector magnitude data alone to estimate the percentage of time that children spend in each respective activity intensity while indoors with acceptable accuracy.

## Figures and Tables

**Table 1:** Performance of two physical activity intensity classifiers for estimating physical activity volume from 3D camera-derived triaxial acceleration data as compared to direct observation.

	Median (IQR)	AUC (95% C.I.)	Sensitivity	Specificity	Median Difference (90% CI)	*p*-Value	
						Δ = 10%	Δ = 20%
**SED (% time)**							
DO	*25* (20)%						
CART	*33* (27)%	0.89 (0.87, 0.91)	81%	81%	14 (−2, 19)%	>0.05	0.01
MCART	*36 (24)%*	0.85 (0.84, 0.89)	80%	76%	16 (5, 22)%	>0.05	>0.05
**LPA (% time)**							
DO	*37* (28)%						
CART	*29* (13)%	0.87 (0.85, 0.90)	66%	90%	−8 (−17, 0)%	>0.05	0.005
MCART	*40 (10)%*	0.83 (0.78, 0.86)	66%	81%	−4 (−12, 10)%	>0.05	0.02
**MVPA (% time)**							
DO	*28* (18)%						
CART	*24* (15)%	0.92 (0.89, 0.93)	71%	95%	−5 (−7, −2)%	0.02	<0.001
MCART	*25 (17)%*	0.88 (0.84, 0.90)	64%	92%	−3 (−9, 0)%	0.01	<0.001

Note: Table 1 shows differences between percentages of time in each activity intensity as determined by direct observation (DO), a Classification and Regression Tree (CART), and a Multiclass CART (MCART). Median difference underestimates are indicated by negative values, and positive values suggest overestimation relative to the ground truth. Nonparametric Two One-Sided Tests show differential statistical equivalence between the directly observed and classifier-estimated percentages of time in each activity intensity at 10% and 20% difference ( ) cut points, with 90% Confidence Intervals (90% CI). Abbreviations: sedentary behavior (SED), light physical activity (LPA), moderate-vigorous physical activity (LPA), proportion of time (% time), receiver operating characteristic area under the curve (AUC), bootstrapped 95% Confidence Interval (95% CI).

## Supplementary Materials

The following are available online at https://www.mdpi.com/1424-8220/20/4/1141/s1, Video S1: KinectVision_DEMO.mov.
